# *Chrysophyllum cainito*: A Tropical Fruit with Multiple Health Benefits

**DOI:** 10.1155/2020/7259267

**Published:** 2020-02-19

**Authors:** Hau V. Doan, Thao P. Le

**Affiliations:** Department of Pharmacy, School of Medicine and Pharmacy, Tra Vinh University, Tra Vinh 940000, Vietnam

## Abstract

*Chrysophyllum cainito* is a tropical fruit tree with multiple benefits to human health. *C. cainito* possesses strong antioxidant properties either *in vitro* or *in vivo*. Extracts from the leaves, stem bark, fruits, peel, pulp, or seed of *C. cainito* are promising candidates in traditional medicine for curing diabetes and fighting against bacterial, fungal, and viral infections. *C. cainito* leaf extract alone or in a complex formula exhibits anti-inflammatory responses by reducing hypersensitivity, acts as inflammatory markers, and has antinociceptive effects. The leaf extract also increases wound healing speed and assists in regulating fat uptake. In addition, the *C. cainito* fruit shows anticancer activity against osteosarcoma. In conclusion, the aerial parts of *C. cainito* have strong beneficial biological effects on human health.

## 1. Introduction

*Chrysophyllum cainito* L. (*C. cainito*) is a tropical tree belonging to the family Sapotaceae. *C. cainito* is grown as an ornamental or orchard tree, which produces fruit. There are different names for *C. cainito* depending on where it is present such as star apple, cainito, and caimito [1]. The seed of *C. cainito* is in the center which has the appearance of an asterisk when cut transversely, giving the fruit its common English name “star apple.” This tree species is commonly found in Mexico, Argentina, Peru [2], the Pacific side of Guatemala [1], and Vietnam, India, China, Malaysia, and other countries where the altitude is low to medium [3].

Medicinal plants have been used in traditional healthcare systems for years, and *C. cainito* is not an exception. Traditional practicians and patients have used the tree and its fruit as folk medicine. Traditional medicinal plants play vital roles not only in disease prevention but also in treatments [4]. The biological activity of *C. cainito* has been researched heavily lately, which can be seen by the overall health benefits and properties of *C. cainito* being studied which includes but is not limited to antioxidants [5, 6], antidiabetic [7, 8], anti-inflammatory [9], anticancer [10], and antihypertensive properties [11]. This review is to summarize the valuable information of *C. cainito* where medicinal benefits have been proven.

## 2. Methods

### 2.1. Literature Search Strategy

We searched international electronic databases from ScienceDirect, PubMed Central, Google Scholar, and general Google search tools. Search terms included “Chrysophyllum cainito” or “Chrysophyllum cainito biological activity,” or “cainito health benefits,” or “Chrysophyllum cainito extract,” or “star apple.” We also used the information from the *Vietnamese* book to get the first overview of the species.

### 2.2. Exclusion Criteria

The literature search was conducted with the exclusion of language other than English. The local publish of the national conferences or research articles without a convenient approach and the articles studying about ecosystem, economic, and agricultural aspects of *C. cainito* were also excluded.

## 3. Results

### 3.1. Botanical Profile

*C. cainito* L. is a tropical tree belonging to the family Sapotaceae. *C. cainito* has many common names depending on its geographical location such as golden leaf, West Indian star apple, caimito, star apple, cainito (English), kaimito (Filipino), caïmitier à feuilles d'or, caïmitier, caïmite franche, caïmite des jardins, caimite, bon caïmite, pomme surette, grand caïmite (French), Sino-Tibetan (nam^2^ nom) (Lao), sata apoen (Thai), or vú sũa (the Vietnamese literal translation: breast milk) [1, 12]. *C. cainito* appears as an ornamental tree and produces edible fruits [13].

**Figure 1:**
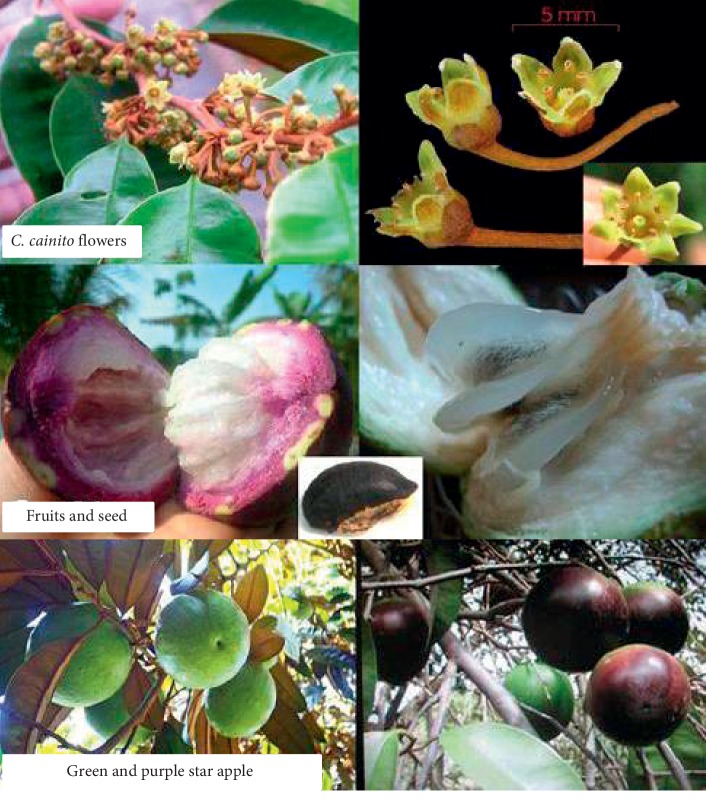
*Chrysophyllum cainito* flowers and fruits.

As described by Morton [14], the star apple tree is an erect tree, with an average height of 8 to 30 meters, with a trunk diameter average of 1 meter. The tree typically has a number of brown-hairy branchlets, with white, gummy latex present inside. The leaves are nearly evergreen with an oblong-elliptic shape, slightly leathery to the touch, rich green, and glossy on the upper surface, coated with silky, with golden-brown pubescence beneath. The morphology of star apple is shown in Figure 1.

Star apple flowers are small, inconspicuous, clustered, greenish-yellow to yellow, or purplish-white with tubular, 5-lobed corolla, and 5 or 6 sepals. The fruit is usually round, oblate, ellipsoid, and 5–10 cm in diameter. The fruits' color may be red-purple, dark-purple, or pale-green. The pulp is soft, white, milky, and sweet and may have up to 10 flattened hard seeds, black at first, with a light area on the ventral side, and turn to light-brown when dry [2].

### 3.2. Distribution

It is commonly known that the origin of the species of *C. cainito* is Central America [2, 3]. However, others say that it may have originated in the West Indies. The tree is well distributed from the south of Mexico to northern Argentina and Peru where the altitudes are relatively low to medium [2] and is planted abundantly on the Pacific side of Guatemala [1]. Vo reported that star apple is omnipresent in Vietnam and has spread across India, China, Sri Lanka, Malaysia, and Indonesia [3].

*C. cainito* is an indigenous species grown in Costa Rica, Cuba, Dominica, Haiti, Honduras, Jamaica, Mexico, the Netherlands Antilles, Nicaragua, Panama, etc. This tree is an alien fruit planted in the United States of America, Uruguay, Venezuela, Argentina, Brazil, Chile, China, Colombia, Peru, Philippines, Thailand, and many nations in Africa such as Egypt, Congo, Ethiopia, South Africa, Mozambique, and Zimbabwe [12].

### 3.3. Nutritional Facts

The flesh of *C. cainito* is very sweet mainly due to the high levels of glucose, which is presented in Table 1 along with further nutritional information. The seeds contain 1.2% of the bitter, cyanogenic glycoside, lucumin, and a number of active compounds. As shown in Table 1, the star apple fruit consists of approximately 78.4–85.7% water. Each 100 g of fruit has 0.72–2.33 g of protein present, 14.65 g of carbohydrates, and between 8.45 and 10.39 g of total sugars. Moreover, the fruit also contains several minerals, vitamins, and amino acids [1, 2].

### 3.4. Phytochemical Analysis of *C. cainito*

The extracts of different parts of *C. cainito* contains phenols, alkaloids, flavonoids, steroids, saponins, tannins, and cardiac glycosides found in the stem bark [7] or fruit [15]. In the year 2002, it was the first time volatile constituents of star apple fruit were analyzed in Cuba [16]. This investigation resulted in a list of 120 constituents separated; among them, 104 were either positively or tentatively identified by gas chromatography (GC) and gas chromatography-mass spectrometry (GC-MS). The major constituents found were (E)-2-hexenal, 1-hexanol, limonene, linalool, *α*-copaene, and hexadecanoic (palmitic) acid. The fraction of *C. cainito* fruit extract was subjected to Diaion HP-20SS resin chromatography. The pure compound from semipurified fraction was obtained by H_2_O : methanol (MeOH) extraction, which was then isolated by a Sephadex LH-20 column and identified by high-performance liquid chromatography (HPLC), nuclear magnetic resonance (NMR), and liquid chromatography-mass spectrometry (LC-MS) analysis. The separation resulted in the presence of cyanidin-3-O-b-glucopyranoside, an anthocyanin antioxidant [17]. Luo et al. [6] reported nine characterized flavonoids from *C. cainito* fruit including (+)-catechin, (–)-epicatechin, (+)-gallocatechin, (–)-epigallocatechin, quercetin, quercitrin, isoquercitrin, myricitrin, and gallic acid. In 2011, Kubola et al. [18] found both green and ripe fruits contain vitamin C, phenolic compounds, and flavonoid compounds, such as gallic acid and quercetin.

Other researchers have found that the leaves of *C. cainito* contain gallic acid, galloyl myrecetrin, rutin, quercetrin, myrecetrin, myricetin, quercetin and two triterpenoids *β*-amyrin and lupeol [19], ursolic acid, *β*-sitosterol, lupeol, and gallic acid [20].

The aerial parts of star apples are rich in phenolic content [7] and total flavonoid content [21], and some of the active compounds have been identified. Those components show promise to have strong antioxidant activity and other biological actions.  Table 2 summarizes the phytochemical compound estimated from *C. cainito*.

### 3.5. Antioxidant Capacity of *C. cainito*

Plants are known as an antioxidant reservoir due to rich phytochemical compounds, particularly phenolics [5], flavonoids [26], and alkaloids [27]. An antioxidant agent possesses preventive effects on oxidation processes, ameliorating cellular damage, which is the initial step in the development of cancer and many other diseases [28]. Recently, there has been increasing evidence that *C. cainito* has high antioxidant activity [21–23, 29].

A semipurified water : methanol extract of *C. cainito* fruit showed strong antioxidant activity toward 2,2-diphenyl-1-picrylhydrazyl (DPPH) free radical with IC_50_ = 7.9 ± 0.3 *μ*g/ml. The strong free radical scavenging activity of the pure compound from semipurified fraction was obtained by H_2_O : methanol (MeOH) extraction, and the isolated cyanidin-3-O-b-glucopyranoside, an anthocyanin antioxidant, was evaluated [17]. Using a DPPH assay, Luo et al. [6] demonstrated that the ethyl acetate soluble fraction from fresh edible fruit of *C. cainito* had high free radical scavenging activity with IC_50_ = 22 *μ*g/ml. Luo et al. [6] concluded that quercetin showed highest antioxidant potential. Similarly, Kubola et al. [18] demonstrated the antioxidant activity of *C. cainito* fruits (both green and ripe) with FRAP (ferric reducing antioxidant power), DPPH, and AEAC (ascorbic acid equivalent antioxidant capacity) assays, where high levels of activity may be due to the vitamin C, phenolic compounds, and flavonoid compounds present.

Other parts of the star apple, the peel, and pulp of the fruit provide promising effects for free radical scavenging via DPPH, FRAP, NO, and TBARS examinations [23]. Leaf extract from 4 types of *C. cainito* with ethanol or acetone at different concentrations revealed their scavenging activity against DPPH. Ningsih et al. [21] concluded that 70% ethanol extract of leaf possessed the highest antioxidant property. The crude extract of *C. cainito* stem bark was evaluated for its antioxidant capacity through ABTS, DPPH, and FRAP assays [7]. The leaf extract significantly reduced the level of malondialdehyde in rat liver and kidney homogenates. Rats that received this extract also showed improved levels of internal antioxidant enzyme glutathione, superoxide dismutase activity, and metallothionein in the liver and kidney homogenates, in comparison to a nontreated gamma radiation-induced oxidative stress rat [20].

*C. cainito*possesses strong antioxidant capacities which are demonstrated *in vitro* and *in vivo*. The background of these actions was reliant on the rich antioxidant phenolic content and total flavonoid content. Therefore, *C. cainito* is a promising natural agent for the prevention and treatment of diseases related to an imbalance of antioxidant homeostasis such as diabetes, atherosclerosis, hypertension, ischaemic diseases, Alzheimer's disease, Parkinsonism, cancer, and inflammation.

### 3.6. Antidiabetic Properties of *C. cainito*

*C. cainito* is a tropical fruit tree having many benefits in lessening the symptoms of diseases, including diabetes. Diabetes mellitus is one of the most common metabolic disorders worldwide. To achieve good glycemic control in diabetes, the advice is to combine changes in lifestyle and pharmacological treatment [30]. In addition to synthetic drugs, medical plants are effective and often more affordable while giving less adverse effects [31, 32].

*C. cainito* has been used as a remedy for diabetic patients traditionally for years [14, 33]; however, the evidence of its benefits has only been investigated recently.

The Dida people, in the area of Divo in Côte-d'Ivoire, Ivory Coast, Africa, create a drink from the stem bark of *C. cainito* which is believed to lessen the effects of diabetes and push it into remission [33]. An infusion of the leaves has been used as an antidiabetic agent [1]. To demonstrate the antidiabetic activity of *C. cainito* extract, an experiment with alloxan-induced diabetic rabbits was performed. The diabetic rabbits had a decrease in blood glucose after drinking *C. cainito* 20 g/l in 6 weeks from 5 g/l to 1.4 g/l (500 mg/dL to 140 mg/dL). From this study, it was concluded that *C. cainito* leaves have a glucose-lowering effect at doses >10 g/l and appear to be toxic at 30 g/l. The hypoglycemic effect mainly occurs through active constituents such as alkaloids, sterols, or triterpenes [34]. In 2016, Hegde et al. [8] studied the antidiabetic effects of *C. cainito* using both model alloxan and streptozotocin-induced diabetic rats. The hydroalcoholic solution (water : alcohol; 1 : 1) was used as a solvent for hot percolation extraction. The extract at doses 200 mg/kg and 400 mg/kg body weight of rats induced blood glycemic reduction significantly in comparison with untreated diabetic control. The concentration of triglyceride, cholesterol levels, and low-density lipoprotein declined, whereas an increase was found in high-density lipoprotein in the treated diabetic rats when compared with diabetic control. Additionally, the extract was found to help protect the islet cells in the pancreas [8].

The ethyl acetate leaf extract of *C. cainito* in Indonesia significantly decreased blood sugar levels by days 7, 10, and 14 in a chemical-induced diabetic rat model. All doses (25, 50, and 75 mg/kg) showed a decrease in blood sugar levels when compared to the negative control, but still below a metformin control comparison, of which the rats that received 75 mg/kg extract exhibited the most effective result [24]. The mechanisms underlying the antidiabetic property of *C. cainito* were uncovered by the study of Doan et al. [7], where the aqueous crude extract of the stem bark was used to investigate its effect on diabetes. They reported that a single dose of the extract (500 mg/kg) significantly decreased fasting blood glucose level of alloxan-diabetic mice from 387.17 ± 29.84 mg/dl to 125.67 ± 62.09 mg/dl after 6 hours from treatment. The extract could reduce the area under the curve of blood glucose in an oral glucose tolerance test applied on normal mice. To reduce glucose levels, the extract might stimulate the absorption of glucose in muscle tissue over glucose uptake in the intestinal membrane. The *in vitro* study revealed the strong inhibition power of *C. cainito* extract on *α*-glucosidase, a key enzyme in the digestion process of long-chain carbohydrates to an absorbable sugar form. Although the action mechanism of *C. cainito* on diabetic control has not been fully explored, the antidiabetic property of *C. cainito* was confirmed and the topic of this plant has been attracting global scientists.

### 3.7. Antimicrobial and Antiviral Activity of *C. cainito*

The pulp and seed of *C. cainito* were analyzed for their antimicrobial activity using agar well diffusion methods [15]. The seed and pulp exhibited varying levels of antibacterial and antifungal activities against some clinical isolates such as *Staphylococcus aureus*, *Escherichia coli*, *Salmonella typhi*, *Pseudomonas aeruginosa*, *Candida albicans*, *Aspergillus*, and *Penicillium ascomycetous* fungi. The extract was more sensitive to *Staphylococcus*, *Pseudomonas*, and *Salmonella* at concentrations of 250 mg/ml, 250 mg/ml, and 31.25 mg/ml, respectively, where the zones of inhibition ranged from 1 mm to 10 mm [15]. The green synthesis of silver nano/micro particles of leaf extract from star apple combined with leaf extract from mango inhibited the growth of *Staphylococcus aureus* [35], and interestingly, the peels of *C. cainito* exhibited significant antiviral activity. Antiviral activity was demonstrated through HIV-1 RT inhibition using a nonradioactive immuno/colorimetric assay. Extracts from the peels of *C. cainito* showed HIV-1 reverse transcriptase inhibition values of 72.55 ± 2.26% [36]. It can therefore be concluded that *C. cainito* can be considered as a potential source of antibacterial, antifungal, and anti-HIV-1 virus substance.

### 3.8. Antihypertensive Activity of *C. cainito*

The blood pressure lowering ability of *C. cainito* pulp was first discovered by Mao et al. [11]. Firstly, the extracts and fractions from *C. cainito* pulp showed inhibition of the angiotensin-I-converting enzyme (ACE) potential, known as a key part of the renin-angiotensin system that regulates blood pressure [37]. ACE inhibition activity was found to be maximized in ethyl acetate fractions of alcoholic extract, compared to all other fractions. Secondly, an *ex vivo* study using isolated tissue of aorta showed a prominent relaxation effect occurred when treated with the extract or fraction containing the concentration of phenolics equal to 50 *μ*g/ml and 10 *μ*g/ml gallic acid equivalents (GAE) which were comparable with standard control captopril 0.5 and 0.25 *μ*g/ml. Then, finally, an ethyl acetate fraction of alcoholic extract was found to reduce the elevated arterial pressure of a salt-induced hypertensive rat significantly compared to that of a nonhypertensive rat.

### 3.9. Anti-Inflammatory Effect of *C. cainito*

The antihypersensitivity and anti-inflammatory effects of crude methanolic extract (CME), fractions, and two isolated triterpenes (Lup-20(29)-en-3*β*-O-hexanoate and 3*β*-Lup-20(29)-en-3-yl acetate) obtained from the leaves of *C. cainito* on carrageenan-induced hypersensitivity and paw oedema mice were determined [9]. According to the report, hexane fraction (Hx) showed slight, significant inhibition of mechanical hypersensitivity induced by carrageenan, whereas the ethyl acetate fraction showed no effect. CME slightly inhibited paw oedema compared with the control group (*p* < 0.0001). Both the CME and the Hx significantly reduced the myeloperoxidase activity. The chloroform fraction was found to reduce the level of IL-1*β* in the paw tissue with TNF-*α* releasing still intact. The two isolated triterpenes presented inhibition activity on carrageenan-induced hypersensitivity in mice.

In support of this, the methanol extract from *C. cainito* leaf proved the inhibition activity on the phagocytosis and significantly decreased IL-6, TNF-*α*, NO, and H_2_O_2_ released by the macrophage. Also, this study confirmed that the extract had a low cytotoxicity effect against the normal Vero cells [25].

Déciga-Campos et al.[38] stated that the ethanol extract of *C. cainito* leaf in combination with the extract of *Pouteria campechiana* (Kunth) Baehni, *Citrus limonum*, and *Annona muricata* Linn with a ratio of 1 : 1: 1 : 1 demonstrated that each plant displayed antinociceptive potential through formalin and capsaicin tests. Additionally, the formula revealed antihyperalgesic effects in alloxan hyperglycemic rats.

### 3.10. Activities of *C. cainito* in Bone Diseases

Osteosarcoma is one of the most common primary malignant bone cancers and is a disease mainly diagnosed in adolescents and young adults and small percentage of cases have occurred in older adults [39]. The polyphenolic fraction of *C. cainito* pulp with a concentration of 50 *μ*g GAE/ml and higher has shown an increase in reactive oxygen species (ROS) concentration significantly in U-2 osteosarcoma cells compared to the nontreatment control group. The fraction produced ROS statistically equivalent to ROS production stimulated by 100 *μ*M H_2_O_2_. Extracts with 25 to 200 GAE *μ*g/mL showed major linear relations with U-2 osteosarcoma (ATCC HTB-96™) cell death, where the value of half-maximal effective concentration (EC_50_) was 133 GAE *μ*g/ml. The supposed mechanism of cell death was oxidative stress, leading the cells to enter apoptosis and ultimately to cell death thereafter [10].

However, the extract from *C. cainito* leaf modulated the elevation of the vertebral trabecular bone density in an osteoporosis mice model. This report indicated that the 70% ethanol extract of *C. cainito* leaf showed the highest activity with the dose of 400 mg/kg body weight of mice/day. This result was obtained due to active phytoestrogen content in 70% ethanol extract of *C. cainito* leaves [40].

### 3.11. Wound Healing Activity of *C. cainito*

The application of an ethanolic extract of *C. cainito* leaves on excision, soft tissue, and wounds significantly shortens the recovery time of the wound compared to control drugs (Jatyadi Taila and Betadine) [41]. An ethanolic extract of *C. cainito* leaves was prepared as an ointment with three different doses 2.5%, 5%, and 20%. The test ointment (0.5 g each) was used and applied on excision wound sites of rats. The highest dose of the extract (20%) showed the fastest rate of wound healing and complete recovery on the 10th day after wound, whereas the control group showed complete wound recovery on the 12th day after wound. The parameters of hydroxyproline, hexosamine, and proteins are the biomarkers of the rate of wound healing and collagen turnover [42], which were found to be substantially increased in the granulation tissue of treated rats when compared with the untreated control.

### 3.12. Antilipase Activities of *C. cainito*

Obesity leads to an increase in risk for the development of chronic conditions including cardiovascular diseases [43], diabetes [44], hypertension [45] cancer [46], and many others [47]. One of the approaches to control body weight is to inhibit the absorption of fat, and in this case, *C. cainito* is an interesting candidate because its extract can interfere with the activity of lipase [48]. The methanol extract of *C. cainito* leaves could inhibit 74.91% activity of lipase. However, the hexane partition from the methanol extract showed a higher inhibition effect up to 92.11%. It was concluded that the nonpolar pancreatic lipase inhibitory agents dissolve in the hexane solution most efficiently.

## 4. Discussion

The normal level of reactive oxygen species (ROS) in the human body plays a crucial role in physiological and pathophysiological activities such as regulating protein phosphorylation, ion channel, transcription factors, and many biosynthetic processes. In contrast, ROS and free radicals can damage cellular structures, including carbohydrate, nucleic acid, protein structure, and lipids [49]. ROS accumulate daily from both intracellular metabolism and exogenous factors such as stress, ionizing radiation, or toxic. Although there is the antioxidant defense system in the body, excess production of ROS can lead to the development of many chronic diseases such as cancer, diabetes and degenerative diseases (neurodegenerative, aging), or digestive diseases [50]. Many studies suggest that antioxidant agents can ameliorate oxidative damage, which helps to prevent disease progression and improve treatment strategies [51]. Those activities have been found richly in medicinal plants [52–54]. *C. cainito* extracts possessed strong antioxidant potential *in vitro* [7, 21, 22] and *in vivo* [20]. Hence, using *C. cainito* may promote oxidative status in cells and tissues. It is a promising candidate for healing various diseases, in particular, oxidative stress-related diseases.

As described above, multiple pharmacological activities of *C. cainito* have been discovered, including antimicrobial, antiviral, antihypertensive, anti-inflammatory, antilipase activity, wound healing, antibone cancer, and antidiabetic actions. By recognizing multiple biological functions, *C. cainito* has become more interesting to scientists. The effect of *C. cainito* on blood glucose concentration has caught the consideration of scientists most [7, 8, 24, 34].

It is known that diabetes mellitus is a chronic disease characterized by long-term hyperglycemia. The characteristic is derived from insufficient insulin hormone or from the utilization of insulin improperly. Diabetes mellitus has been affected by approximately 415 million people worldwide in 2015, and the number of cases has been estimated to increase to 642 million in 2040 [55]. Most medications for type 2 diabetes are synthetic drugs orally taken, but the frequent insulin injection is essential for type 1 diabetes to survive. Diabetes medications, however, have exhibited many adverse effects such as the sodium-glucose transport protein 2 inhibitor, thiazolidinediones, or sulfonylureas [56–59]. Therefore, antidiabetic agents from natural plants are of interest since natural products are sources of novel drug leads and relatively none or less toxic [60, 61]. *C. cainito* could significantly decrease blood glucose levels in experimental mice [7], rats [24], and rabbits [34]. Beyond glucose regulation, the extract also improved lipid profiles and contributed to pancreatic *β*-cells regeneration [8]. Several mechanisms of the blood glucose lowering effect of *C. cainito* were revealed *in vitro* and *in vivo*, for instance, protecting *β*-cells, stimulating glucose consumption from muscle, inhibiting *α*-glucosidase activity, or basing on its antioxidant power. Unfortunately, the active chemicals in *C. cainito* crude extract have not been explored yet. Therefore, active ingredients within the extract need to be further investigated.

Besides antioxidant and antidiabetic potentials, *C. cainito* also has strong antibacterial, antiviral, and antifungal activities [15]. Its extract could inhibit the growth of common clinical isolated bacterial strains like *S. aureus*, *E. coli*, or *C. albicans*. However, the action mechanisms remain undiscovered. These antimicrobial actions may play a part in the treatment of infectious diseases or used as an alternative antimicrobial agent. Moreover, the antimicrobial capacity of the extract enhances its antidiabetic effect in diabetic patients with infection. Infectious diseases were difficult to fight and relatively more serious in diabetes mellitus due to immune system dysfunction [62].

## 5. Conclusions

Although *C. cainito* is planted worldwide mainly for fruits which contain many nutrients such as proteins, carbohydrates, vitamins, phenolics, and amino acids. Its extract has been used as traditional medicine for a long time, and recently several biological functions of this extract have been explored and demonstrated. Many pharmacological activities of *C. cainito* found such as antioxidant, antidiabetic, anti-inflammation, anticancer, antihypertensive, promoting wound healing, and vertebral trabecular bone cell division. Among them, *C. cainito* exhibits prominently capabilities in antioxidant, antimicrobiological, and antidiabetic activities. Recent evidences support the use of *C. cainito* for traditional users. Nonetheless, the mechanisms underlying all activities and pure and active compounds as well as more applications of this plant are expected to be further studied.

## Figures and Tables

**Table 1:** Nutrient value per 100 g of star apple fruit (edible portion) [2].

Constituents	Value
Water content	78.4–85.7%
Calories	67.2 kcal
Protein	0.72–2.33 g
Carbohydrates	14.65 g
Total sugars	8.45–10.39 g
Fiber	0.55–3.30 g
Ash	0.35–0.72 g
Calcium	7.4–17.3 mg
Phosphorus	15.9–22.0 mg
Iron	0.30–0.68 mg
Carotene	0.004–0.039 mg
Thiamin	0.018–0.08 mg
Riboflavin	0.013–0.04 mg
Niacin	0.935–1.340 mg
Ascorbic acid	3.0–15.2 mg
Tryptophan	4 mg
Methionine	2 mg
Lysine	22 mg
Total volatiles	0.154 mg
Total phenols	217.0–387.1 mg

**Table 2:** Phytochemical compounds of *C. cainito*.

Aerial part	Phytochemical compounds	Methods	Ref.
Fruit	(+)-Catechin; (–)-epicatechin; (+)-gallocatechin; (–)-epigallocatechin; quercetin; quercitrin; isoquercitrin; myricitrin; gallic acid	Vacuum-liquid chromatography	[6]

Fruit	104 volatile constituents with major compounds were (E)-2-hexenal; 1-hexanol; limonene; linalool; *α*-copaene; hexadecanoic acid	GC-MS	[16]

Fruit	Alkaloids; glycosides; proteins and amino acids; sterols; carbohydrates; phenolic compounds; flavonoids; saponins and tannins	Color test	[8]

Fruit	Cyanidin-3-glucoside	Column chromatography	[17]

Fruit	Gallic acid; protocatechuic acid; *p*-hydroxybenzoic acid; chlorogenic acid; syringic acid; *p*-cormaric acid; ferulic acid; sinapinic acid; rutin; myricetin; luteolin; quercetin; apigenin; kaempferol; vitamin C	HPLC-DAD	[18]

Leaf	Lup-20(29)-en-3*β*-O-hexanoate; 3*β*-Lup-20(29)-en-3-yl acetate	Column chromatography	[9]

Fruit	Phytosterols; glycoside; alkaloids; tannins; flavonoids; saponin; protein; amino acid; carbohydrate; fat and fixed oil	Color test	[11]

Leaf	Terpenoids; phenolics; alkaloids; quaternary alkaloids; *n*-oxides; ursolic acid; *β*-sitosterol; lupeol; gallic acid	HPTLC	[20]

Fruit	Vitamin C; anthocyanins; phenolics; flavonoids; carotenoids	Color test followed by UV-Vis	[22]

Pulp	Saponin; tannins; flavonoids; steroid; cardiac glycoside; vitamin C; vitamin A	Color test followed by UV-Vis	[15]

Seed	Flavonoids; steroid; vitamin C; vitamin A	Color test followed by UV-Vis	[15]

Leaf	Flavonoids; phenolics	Color test followed by UV-Vis	[21]

Fruit	Polyphenolic compounds	HPLC-PAD	[23]

Stem bark	Phenols; tannins; glycosides; terpenoids and saponin	Color test	[7]

Leaf	Flavonoids; saponin	Color test	[24]

Leaf	Lupeol acetate; alpha-amyrin acetate	GC-MS	[25]

Leaf	Gallic acid; 3′ galloyl myrecetrin; rutin; quercetrin; myrecetrin; myricetin; quercetin; *β*–amyrin; lupeol	GC-MS	[19]
